# Systematic Screening of Commonly Used Commercial Transfection Reagents towards Efficient Transfection of Single-Stranded Oligonucleotides

**DOI:** 10.3390/molecules23102564

**Published:** 2018-10-08

**Authors:** Tao Wang, Leon M. Larcher, Lixia Ma, Rakesh N. Veedu

**Affiliations:** 1 Centre for Comparative Genomics, Murdoch University, Perth, WA 6150, Australia; Tao.Wang@murdoch.edu.au (T.W.); LeonMaria.Larcher@murdoch.edu.au (L.M.L.); 2 Perron Institute for Neurological and Translational Science, Perth, WA 6009, Australia; 3 School of Statistics, Henan University of Economics and Law, Zhengzhou 450046, China; realmlx@163.com

**Keywords:** single-stranded oligonucleotide, transfection reagent, cationic lipid, gene transfection, cytotoxicity

## Abstract

Non-viral vector-mediated transfection is a core technique for in vitro screening of oligonucleotides. Despite the growing interests in the development of oliogonucleotide-based drug molecules in recent years, a comprehensive comparison of the transfection efficacy of commonly used commercial transfection reagents has not been reported. In this study, five commonly used transfection reagents, including Lipofectamine 3000, Lipofectamine 2000, Fugene, RNAiMAX and Lipofectin, were comprehensively analyzed in ten cell lines using a fluorescence imaging-based transfection assay. Although the transfection efficacy and toxicity of transfection reagents varied depending on cell types, the toxicity of transfection reagents generally displayed a positive correlation with their transfection efficacy. According to our results, Lipofectamine 3000, Fugene and RNAiMAX showed high transfection efficacy, however, RNAiMAX may be a better option for majority of cells when lower toxicity is desired. The transfection efficacy of Lipofectamine 2000 was compromised by its high toxicity, which may adversely affect its application in most cells. We firmly believe that our findings may contribute to the future In vitro delivery and screening of single-stranded therapeutic oligonucleotides such as antisense oligonucleotides, antimiRs, and DNAzymes.

## 1. Introduction

Nucleic acid-based therapeutics have received significant attention in recent years for the treatment of several diseases, however, the delivery of therapeutic nucleic acids still remain a major challenge [1]. Although an effective in vivo transfection strategy for single-stranded oligonucleotide (SSO) molecules such as antisense oligonucleotide (ASO), antimiR and DNAzyme is not available, as a standard practice, various transfection reagents have been employed for their in vitro screening [2,3]. Double stranded plasmid DNAs can be transfected effectively by using high efficient viral vectors, but, non-viral vectors are generally used for screening single-stranded oligonucleotide in vitro. However, despite the pivotal role of non-viral transfection reagents playing in SSO screening, a comprehensive comparison of the transfection efficacy of current commercially available transfection reagents has not been reported, although they are generally compatible with different types of genetic materials, including ASO, siRNA, antimiR, and plasmids. Previous comparative studies of transfection reagents have mainly focused on plasmid DNA constructs [4,5,6]. The transfection efficiency of non-viral transfection reagents is not only associated with cell type, cell and media conditions, but also the type of nucleic acid molecule [7,8]. Herein, we report the transfection efficiency and toxicity comparison of commonly used commercial transfection reagents for in vitro delivery of SSOs in ten established cell lines, to assist researchers working in this rapidly progressing field of oligonucleotide therapeutic development.

## 2. Results

### 2.1. Method to Measure the Transfection Efficacy of SSO

**Figure 1:**
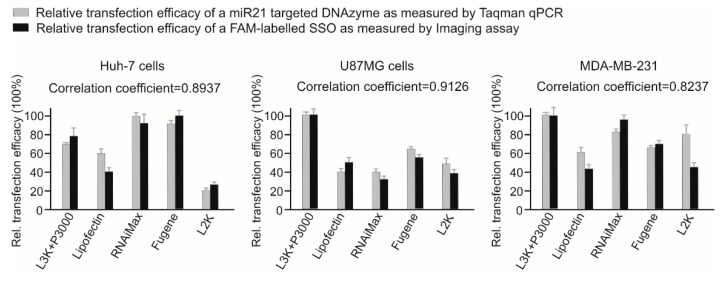
Comparison of two methods for efficacy assay of different transfection reagents using SSO. This assay was conducted by two independent experiments as indicated. The data from both tests were normalized by setting the highest transfection data as 100%. L3K: Lipofectamine 3000, L2K: Lipofectamine 2000.

Ideally the efficacy of a transfection reagent for SSO transfection should be measured by functional assays such as western blotting or qPCR to test the gene regulation effect of SSO at the RNA or protein levels. To facilitate this comprehensive comparison, we adopted a simple and effective approach to investigate the transfection efficacy of different transfection reagents by fluorescence imaging and quantifying the cellular fluorescence of a FAM-labelled SSO sequence. This is based on the hypothesis that, the chance of SSO to meet their intracellular targets in cytoplasm/nucleus would depend mainly on the amount of oligonucleotide delivered to cells [9]. To confirm this, first we tested the transfection efficacy of a 24-mer DNA-SSO (miR21 targeting DNAzyme) in three different cell lines including Huh-7 cells, U87MG cells, MDA-MB-231 cells, by incubating with different transfection reagents in line with the manufacturer’s recommendations. After 24 h, total miRNAs from different treatments were collected, followed by performing a TaqMan qPCR assay to measure the expression of the miR21 target. As demonstrated in Figure 1, the imaging assay results showed same trend with the functional assay, with correlation coefficients of 0.8937, 0.9126, 0.8237 for Huh-7, U87MG and MDA-MB-231 cells, respectively.

### 2.2. Evaluation of Transfection Reagent (TR)/SSO Ratio

**Figure 2:**
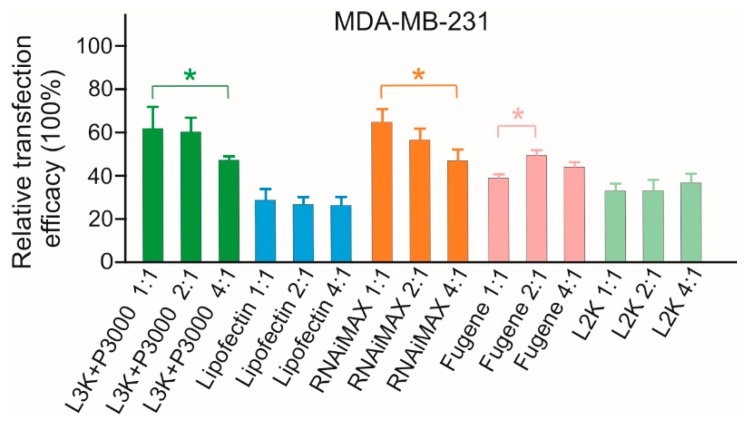
In vitro transfection efficiency of transfection reagent (TR)/SSO complexes at different ratios (*v*/*w*) in MDA-MB-231 cells. Cells were transfected with TR/DNA complexes in the absence of serum with three different amounts of SSO. L3K: Lipofectamine 3000, L2K: Lipofectamine 2000. * p < 0.01.

Before conducting the transfection analysis in the selected cell lines, the transfection conditions were evaluated first with MDA-MB-231 cells by using 3 µL transfection reagent/500 µL medium in 24-well plates (the amount of transfection reagent was within the manufacturer’s suggested dose range for all reagents under test) while varying the amounts of the SSO sequence at different TR/SSO ratios in order to form the complexes. As demonstrated in Figure 2, the variation of SSO generally did not show a significant difference in the transfection efficiency, but Lipofectamine 3000, RNAiMAX and Fugene displayed slight differences. This result is consistent with a previous comparison study performed on plasmid DNA [4]. Considering TR/SSO ratio of 2:1 did not show significant differences among the treatment groups for all six transfection reagents, this ratio (3 µL TR plus 1.5 µg ASO/500 µL medium in a 24-well plate) was used in all subsequent transfection studies. 

### 2.3. Comparative Analysis of the Transfection Efficacy and Cytotoxicity in Ten Cells

#### 2.3.1. Huh-7 Liver Cancer Cells

**Figure 3:**
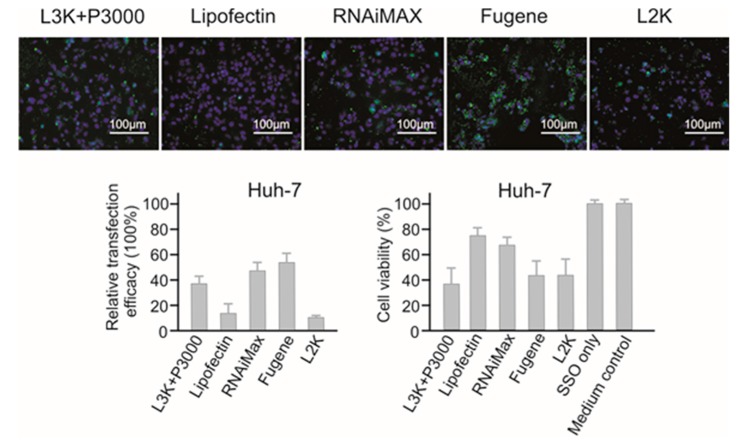
In vitro transfection efficiency and cytotoxicity of different transfection reagents in Huh-7 cells. L3K: Lipofectamine 3000, L2K: Lipofectamine 2000.

Out of the five transfection systems tested, Fugene and RNAiMAX demonstrated the highest relative transfection efficacy in Huh-7 cells (55.42% and 46.12% respectively) compared to other tested reagents (Figure 3). Lipofectamine 3000 showed the third highest transfection efficacy (37.02%). Lipofectamine 2000 showed the lowest transfection efficacy, with 8.91% relative transfection efficacy, approximately 6 folds lower than RNAiMAX. However, the highest transfection efficacy of Fugene was compromised by high cytotoxicity, with 40.74% cell viability as measured by MTT assay, similar with that of Lipofectamine 3000 and Lipofectamine 2000. Lipofectin and RNAiMAX showed lower cytotoxicity to Huh-7 cells, with 75.34% and 67.25% cell viability respectively. If the toxicity is an important factor to consider, RNAiMAX would be a better reagent for Huh-7 cells, otherwise, Fugene performed better in terms of transfection. 

#### 2.3.2. SHSY5Y Neuroblastoma Cells

**Figure 4:**
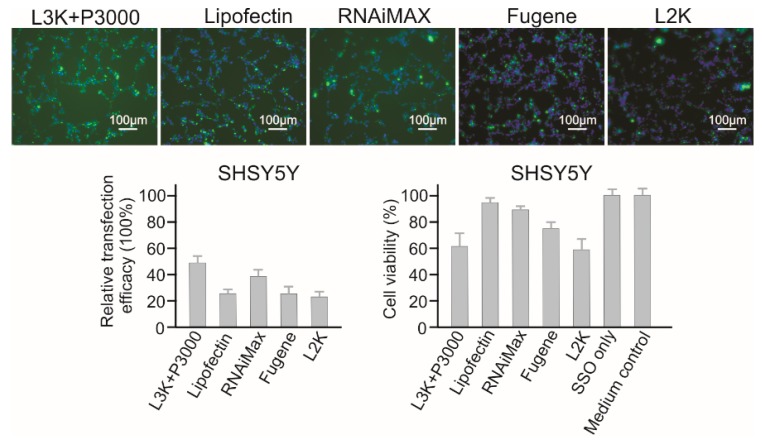
In vitro transfection efficiency and cytotoxicity of different transfection reagents in SHSY5Y cells. L3K: Lipofectamine 3000, L2K: Lipofectamine 2000.

As shown in Figure 4, for SHSY5Y cells, Lipofectamine 3000 and RNAiMAX showed better transfection efficacy (47.17% and 37.26%, respectively). Lipofectin (26.40%) displayed similar transfection efficacy to Fugene (24.07%) and Lipofectamine 2000 (22.21%). As for cytotoxicity, this cell line displayed a better resistance to most reagents, except Lipofectamine 3000 (61.01% viability) and Lipofectamine 2000 (59.14% viability). RNAiMAX displayed both good transfection efficacy and low cytotoxicity on SHSY5Y cells, with the viability of 90.74%. Although Lipofectamine 3000 displayed the highest transfection efficacy, its high cytotoxicity effect poses a concern for functional studies. 

#### 2.3.3. HepG2 Liver Cancer Cells

**Figure 5:**
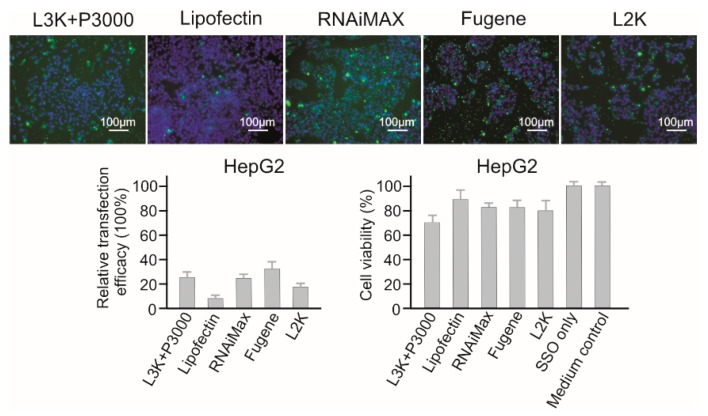
In vitro transfection efficiency and cytotoxicity of different transfection reagents in HepG2 cells. L3K: Lipofectamine 3000, L2K: Lipofectamine 2000.

Lipofectamine 3000, RNAiMAX and Fugene displayed similar transfection efficacy to HepG2 cells (25.44%, 24.32%, and 32.50%, respectively, Figure 5). Although Lipofectin showed the lowest toxicity to HepG2 cells (89.54% viability), it displayed the lowest transfection efficacy too (8.29%). Generally, HepG2 cells displayed resistance to the toxicity of majority of the reagents tested, with Lipofectamine 3000 (70.59 % viability), being the most toxic reagent. For HepG2 cells, Fugene could be recommended considering its high transfection efficacy and low cytotoxicity (82.64%). 

#### 2.3.4. JU77 Lung Mesothelioma Cells

**Figure 6:**
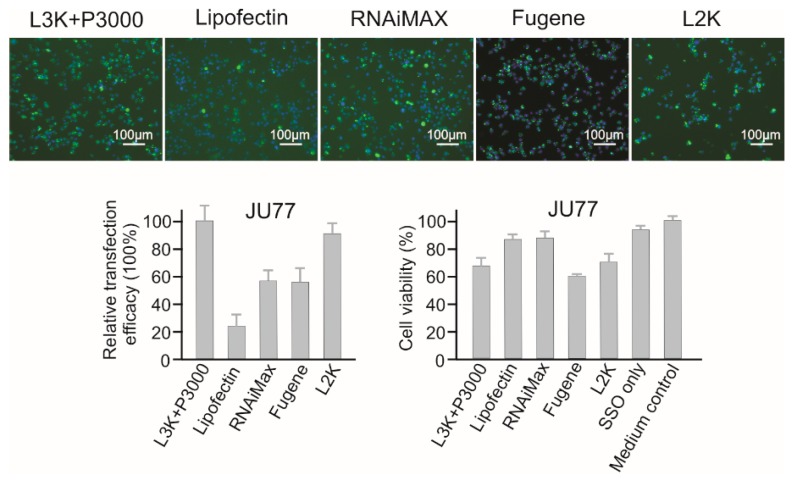
In vitro transfection efficiency and cytotoxicity of different transfection reagents in JU77 cells. L3K: Lipofectamine 3000, L2K: Lipofectamine 2000.

JU77 cells were relatively easy to transfect (Figure 6). Lipofectamine 3000 showed the highest transfection efficacy (normalized as 100%) in this study, followed by Lipofectamine 2000 (90.21%), but these two transfection reagents also displayed high cytotoxicity to JU77 cells (68.21% and 69.27% viability). The transfection efficacies of RNAiMAX and Fugene were very similar, with medium high efficacy of 57.86% and 55.12%. However, differing from the good safety profile displayed by RNAiMAX (87.13% viability), Fugene showed the most toxic effect, with cell viability of 60.08%. Although the treatment of Lipofectin was quite safe (86.90% viability), it did not show high transfection efficacy (23.75%). 

#### 2.3.5. HL60 Promyelocytic Leukemia Cells

**Figure 7:**
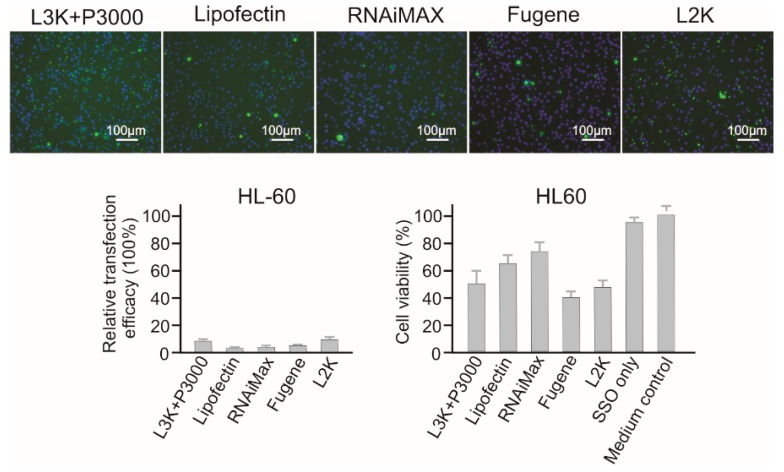
In vitro transfection efficiency and cytotoxicity of different transfection reagents in HL60 cells. L3K: Lipofectamine 3000, L2K: Lipofectamine 2000.

As a type of suspension cell, HL60 cells displayed strong resistance to all the tested transfection reagents. As shown in Figure 7, only Lipofectamine 3000 (8.93%) and Lipofectamine 2000 (7.89%) displayed transfection at very low levels, and that was accompanied by high cytotoxicity, with 51.89% and 46.21% cell viability for Lipofectamine 3000 and Lipofectamine 2000, respectively. Other reagents did not demonstrate effective transfection to this cell line.

#### 2.3.6. Primary Muscle Myoblasts

**Figure 8:**
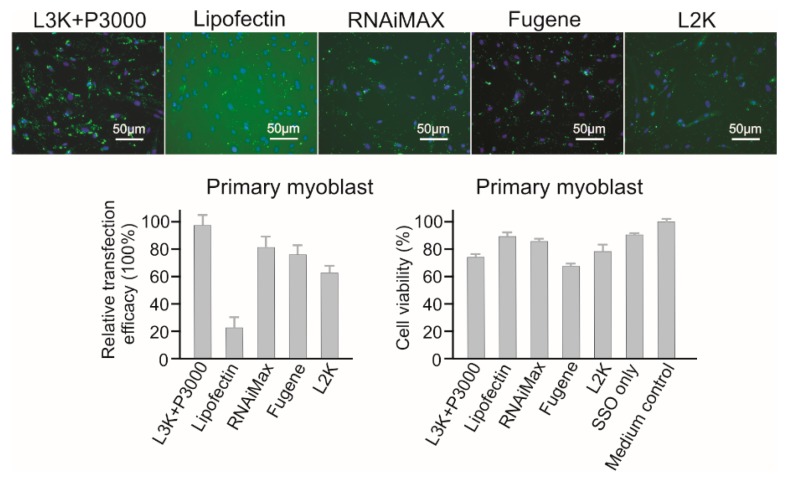
In vitro transfection efficiency and cytotoxicity of different transfection reagents in Primary myoblast cells. L3K: Lipofectamine 3000, L2K: Lipofectamine 2000.

Lipofectamine 3000, RNAiMAX, Fugene and Lipofectamine 2000 all displayed high transfection efficacy (97.78%, 80.34%, 77.86% and 60.01%, respectively) to primary myoblast cells (Figure 8). Among them, although RNAiMAX displayed the second highest transfection efficacy, and it had the best safety profile, with 83.65% cell viability. Fugene was found to be the most toxic reagent for this cell line, with a viability of 68.45%. Again, the good safety profile (89.92% viability) of Lipofectin was accompanied by low transfection efficacy (22.33%). Therefore, for primary myoblast cells, Lipofectamine 3000 or RNAiMAX could all be considered. 

#### 2.3.7. HEK293 Embryonic Kidney Cells

**Figure 9:**
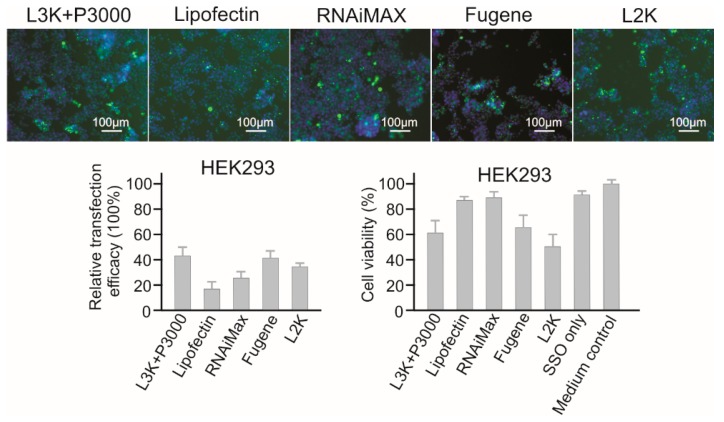
In vitro transfection efficiency and cytotoxicity of different transfection reagents in HEK293 cells. L3K: Lipofectamine 3000, L2K: Lipofectamine 2000.

For HEK293, Lipofectamine 3000, Fugene and RNAiMAX showed the highest transfection efficacies (42.52%, 40.51%, and 25.60%, respectively) (Figure 9). However, unlike Fugene and Lipofectamine 3000 that displayed strong toxicity to HEK293 cells (65.83% and 61.70% viability), RNAiMAX showed the highest cell viability of 89.30%, followed by Lipofectin (87.29%). Lipofectamine 3000, Fugene and RNAiMAX could be used for screening SSO in HEK293 cells. 

#### 2.3.8. MCF-7 Breast Cancer Cells

**Figure 10:**
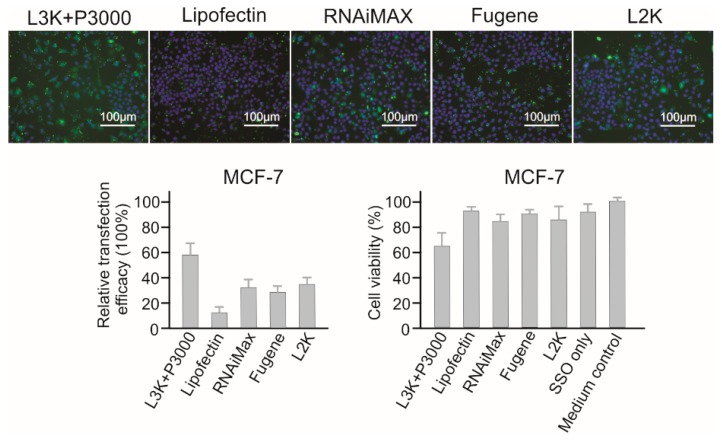
In vitro transfection efficiency and cytotoxicity of different transfection reagents in MCF-7 cells. L3K: Lipofectamine 3000, L2K: Lipofectamine 2000.

As for MCF-7 cells (Figure 10), Lipofectamine 3000 demonstrated the highest transfection efficacy (58.13%), followed by Lipofectamine 2000 (33.29), RNAiMAX (31.92%) and Fugene (27.80). Although Lipofectin showed the best safety profile with a cell viability of 92.49%, it did not show prominent transfection efficacy (6.62%). Generally, MCF-7 cells are resistant to transfection reagents mediated cell damage, and only Lipofectamine 3000 treatment (62% cell viability) displayed a lower than 80% cell viability. 

#### 2.3.9. MDA-MB-231 Breast Cancer Cells

**Figure 11:**
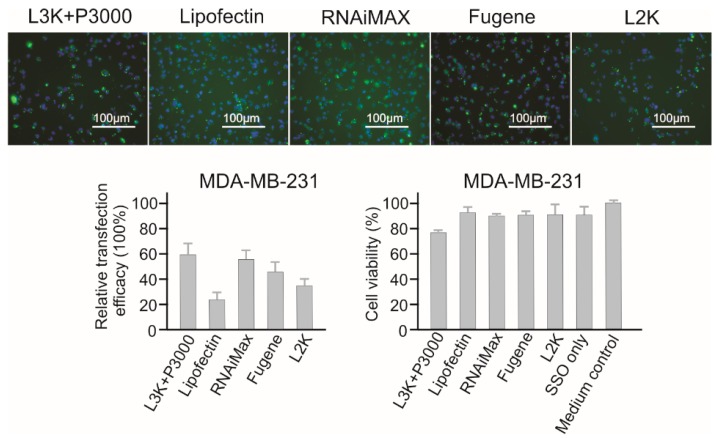
In vitro transfection efficiency and cytotoxicity of different transfection reagents in MDA-MB-231 cells. L3K: Lipofectamine 3000, L2K: Lipofectamine 2000.

Similar to MCF-7 cells, Lipofectamine 3000, RNAiMAX and Fugene showed the highest transfection efficacy in MDA-MB-231 cells, with 59.32%, 55.49% and 43.92% respectively (Figure 11). 

While Lipofectin (23.73%) did not perform well, Lipofectamine 2000 (33.08%) displayed a medium high transfection efficacy. Again, similar to MCF-7 cells, MDA-MB-231 displayed low sensitivity to transfection reagents mediated cell damage, with Lipofectamine 3000 (78.37% cell viability) displaying the lowest cell viability. 

#### 2.3.10. U87MG Brain Cancer Cells

**Figure 12:**
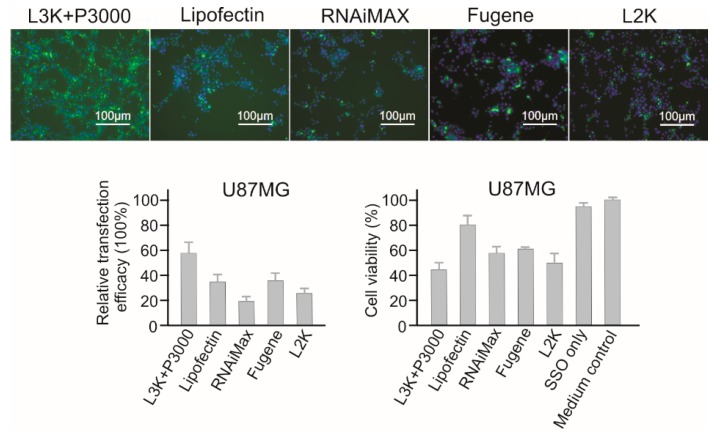
In vitro transfection efficiency and cytotoxicity of different transfection reagents in U87MG cells. L3K: Lipofectamine 3000, L2K: Lipofectamine 2000.

Generally, U87MG is vulnerable to majority of the transfection reagents. As shown in Figure 12, the highest transfection efficacy of 58.21% was recorded for Lipofectamine 3000. Only Lipofectin (38.64% transfection efficacy) recorded a higher than 80% cell viability. Therefore, both Lipofectamine 3000 and Lipofectin are suggested for U87MG cells. 

### 2.4. High Transfection Efficacy Generally Accompanied by High Cytotoxicity

**Figure 13:**
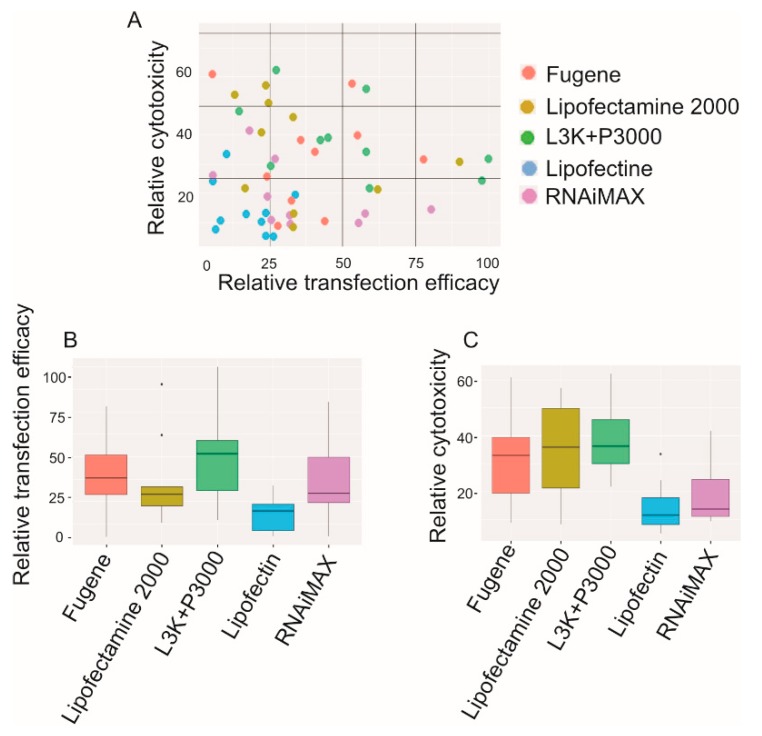
Relative transfection and cytotoxicity patterns of different transfection reagents. (**A**) the overall transfection efficacy and toxicity of each transfection reagent; (**B**) the relative transfection efficacy of each reagent, demonstrated as median; (**C**) the relative cytotoxicity of each reagent, demonstrated as median. L3K: Lipofectamine 3000.

According to the statistical analysis displayed in Figure 13, it appears that the efficacy of different transfection reagents does display a positive correlation with their cytotoxicity (higher toxicity being accompanied by higher transfection efficacy). According to Pearson’s product-moment correlation calculation, up to 0.7588 correlation coefficient between relative cytotoxicity and transfection efficacy was recorded. Although Lipofectamine 3000 demonstrated the highest transfection efficacies in most cell types, it also shared the highest overall cytoxicity with Lipofectamine 2000. And as demonstrated, Lipofectamine 2000 got up to 90.21% and 62.02% transfection efficacy in JU77 and Primary myoblast cells, respectively, but the average transfection efficacy was only 25.13%, half of that of Lipofectamine 3000. With a relatively low cytotoxicity of 20%, RNAiMAX displayed a relatively high transfection efficacy. On the other side, Fugene recorded the second highest transfection efficacy (35.23% in average), but at the same time displayed high cytotoxicity (34.78% toxicity), similar to that of Lipofectamine 3000 (37.23%) and Lipofectamine 2000 (38.97%). 

### 2.5. SSO Increases the Cytotoxicity of Transfection Reagents

**Figure 14:**
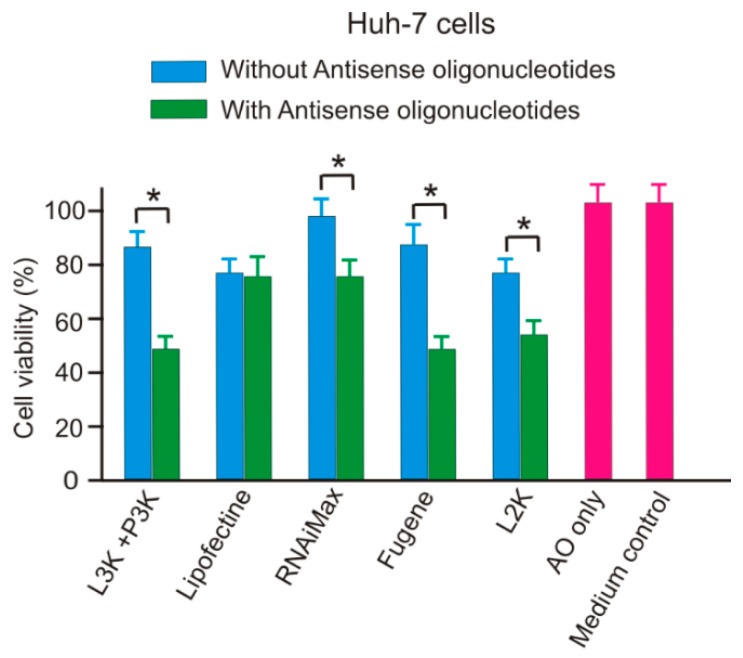
Addition of ASO significantly increase the cytotoxicity of transfection reagents in Huh-7 cells. L3K: Lipofectamine 3000; P3K: P3000, L2K: Lipofectamine 2000. * *p* < 0.01.

Addition of SSO-transfection reagents complex generally increased the cytotoxicity of transfection reagents compared with the naked transfection reagents, as demonstrated in Figure 14. Among the five reagents tested, only Lipofectin did not display significant difference in terms of cytotoxicity. Since the SSO sequence used in this project did not match any human gene sequence, the cause of enhanced cytotoxicity was unlikely by the SSO itself. This result suggests that the interaction of SSO sequences and transfection reagents could increase the toxicity of the tested transfection reagents. 

### 2.6. Transfection Efficacy and Toxicity of Lipofectamine 3000 both Depend on the Addition of P3000

**Figure 15:**
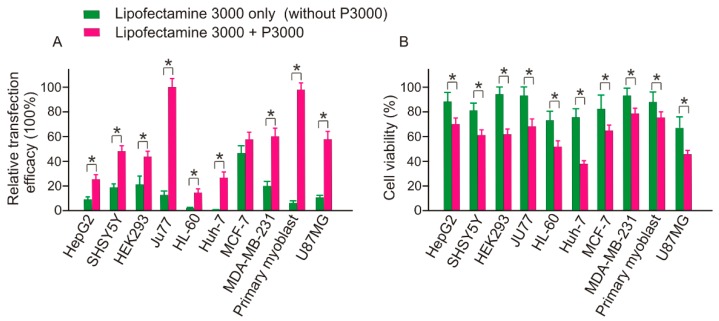
Transfection efficacy and toxicity of Lipofectamine 3000 both depend on the addition of P3000. * *p* < 0.01.

As demonstrated by Figure 15, both the transfection efficacy and cytotoxicity of Lipofectamine 3000 are dependent on the addition of P3000 component. For example, in HEK293 cells, although the addition of P3000 doubled the transfection efficacy from 19.76% to 42.52%, the toxicity was dramatically increased, with the cell viability decreasing from 95.31% to 61.70%. In general, except in MCF-7 cells, which showed a 45.02% transfection efficacy, similar to the group of Lipofectamine 3000 & P3000 treatment, the treatment of Lipofectamine 3000 alone recorded low transfection efficacy in most cases, with minor transfection efficacy recorded in HL60 (2.03%) and Huh-7 (4.45%) cells. Therefore, based on this observation low cytotoxicity, it is not suggested to use Lipofectamine 3000 alone for ASO transfection. 

## 3. Discussion

Previously reported two comparison studies of transfection reagents focused mainly on plasmid DNA delivery by choosing the transfection reagents according to the types of chemical formulations [5,6]. For example, Yamano et al. used six reagents with different formulations including lipo-polymeric (Arrest-In), cationic polymer (ExpressFect), lipid with other components (Fugene), linear polyethylenimine (jetPEI), cationic lipid (Lipofectamine 2000), and activated-dendrimer (SuperFect) [6]. However, it is hard to compare different type of transfection formulation by analyzing data derived from just one representative from each of those formulations. In this study, instead of analysing different types of transfection reagents, as displays in the Supplementary Figure S1, we focused directly on the most commonly used transfection reagents used for SSO transfection in recently published literature, i.e., Lipofectamine 3000, Lipofectamine 2000, RNAiMAX, Lipofectin, and Fugene, to provide practical references for researchers in the field of oligonucleotide therapeutic development. To facilitate this study, we introduced a simple but efficient approach to monitor the efficacy of transfection reagents by fluorescence imaging and quantifying the cellular fluorescence of a FAM-labelled SSO sequence. This is based on the hypothesis that, the chance of SSO to meet their intracellular target in the cytoplasm/nucleus depends mainly on the amount of oligonucleotide delivered to cells. This hypothesis was confirmed by our initial functional assay by using a 24-mer DNAzyme designed to target and inhibit miR21 in Huh-7 cells. The non-specifically bound cell surface oligonucleotides will not contribute to any functional transfection. Methods being able to quench cell surface signals should be used to eliminate the background noise caused by such non-specific binding. During our preparative tests, it was confirmed via a trypan blue method [10] that differing from incubating cells with free fluorescence-labelled oligonucleotides, incubating cells with oligonucleotide/liposome complex (with ratio of 1.5 µg/3 µL transfection reagent in 500 µL) does not display noticeable cell surface fluorescence, As shown in Figure 1, without using fluorescence quenching methods, the data derived from the imaging assay highly correlated with the functional analysis of miR21 expression. As a type of negatively charged oligonucleotide with molecular weight of approximately 6000 to 10,000 Da, SSOs are generally unable to cross the cell membrane effectively [11]. Current chemical transfection reagents, whether it is lipid based or polymer-based formulation, the TR/nucleic acid complexes are typically internalized into cells by endocytosis [12]. After endocytosis, the encapsulated nucleic acid needs to escape from the endosome and release into the cytoplasm to meet their mRNA or miRNA targets or pre-mRNA targets in nucleus. Because oligonucleotides are able to continuously shuttle between the nucleus and the cytoplasm through passive diffusion and active transport [9], once SSOs are internalised, escaping from endosomes becomes a rate limiting step. To this day, although the detailed mechanism of endosome escape is still unclear [3,13], it is conceivable to assume that for the same cell type, the amount of SSOs functionally react with their cytoplasm/nucleus targets should closely correlate with the amount of internalised SSO molecules. Therefore, conjugating SSO with fluorescent dyes and measuring the relative fluorescence intensity provides a simple way to monitor the efficacy of transfection reagents studied. 

According to our experiment, Lipofectamine 3000 and Fugene showed high levels of transfection efficacies in most cell types, but relatively high cytotoxicity poses an important concern when such reagents are considered. Consistent with previous studies [14,15], RNAiMAX, a reagent designed for double stranded siRNA delivery, performed quite well for SSO transfection. As demonstrated, the relatively high transfection efficacy of RNAiMAX accompanied by a relatively low toxicity. On the other hand, Lipofectin did not look suitable for high efficiency SSO transfection in most cell types. Importantly, despite Lipofectamine 2000 being widely used in many published SSO researches, and obtained acceptable transfection efficacy, it displayed the highest toxicity in most cell types tested. The transfection efficacy and toxicity of transfection reagents are highly cell-dependent, and Lipofectamine 3000 and Fugene showed the highest transfection efficacy in most cells, but if low toxic transfection is required, RNAiMAX could be a potential option.

Cytotoxicity is one of the major concerns for transfection reagent selection, especially when subsequent functional assays are scheduled. This is because the toxicity of transfection reagents could nonspecifically activate/inactive certain genes and affect the experimental read-out [16]. Although it has become a common practice to evaluate the toxicity of transfection reagents by conducting parallel experiments using transfection reagents alone, in this study, we found that the toxicity of empty reagents may not faithfully reflect the effect of the mixture of TR/SSO complexes, consistent with a previous study [17]. As the SSO used in this study does not match with any human gene, i.e., the SSO itself does not display any biological effect, suggesting that an increased cytotoxicity of transfection reagents occurred after SSO complexation. Interestingly, differing from what was observed in this work, several previous studies reported an attenuation of toxicity of the transfection reagents through complexation with nucleic acid [18,19]. The reasons for the different observation between our observation and these two publications need to be further studied, but it may due to: (1) difference in the type of genetic material. Unlike the short SSO used in this paper, all of the three publications were conducted with long double stranded plasmid DNAs; (2) Difference in transfection reagents formulation. The cationic lipid, Lipofectamine 3000 comes with two components, Lipofectamine 3000 and P3000. As suggested by the manufacturer’s specification, to transfect cells with double stranded siRNA, it is suggested not to add P3000 reagent. However, apart from siRNA transfection, addition of P3000 component is recommended for DNA samples. However, Lipofectamine 3000 alone has been used in a couple of transfection tests including plasmid and miR mimic [20]. For instance, in a recent study, Bernard and colleagues achieved successful miR217 mimic transfected to HEK293 cells via Lipofectamine 3000 only treatment [20]. Similarly, in this study, we achieved an acceptable transfection efficacy (18.76%) in the same type HEK293 cells via Lipofectamine 3000 only (omitting P3000) treatment. Indeed, the single use of Lipofectamine 3000 represents a great advantage over the combinational use of Lipofectamine 3000 and P3000 by reduced toxicity. As clearly demonstrated in Figure 15, in all the tested cell types, the addition of P3000 contributed to an increase in cytotoxicity of Lipofectamine 3000. However, it appears that omitting P3000 is not a reliable method for SSO delivery. Although the addition of P3000 did not show much difference in transfection efficacy in MCF-7 cells, reduced transfection efficacy was observed in all other cell types when P3000 was omitted, with the transfection efficacy nearly eliminated in HL-60 and Huh-7. Therefore, despite displaying low cytotoxicity, Lipofectamine 3000 alone is generally not suitable for SSO transfection.

In summary, in this work, we comprehensively compared the transfection efficacy of commonly used commercial transfection reagents. The findings will not only contribute to the future In vitro screening of SSO, but also provide references for In vitro delivery of other types of genetic materials such as plasmids and siRNA. 

## 4. Materials and Methods 

### 4.1. Transfection Reagents and SSO

RNAiMAX transfection reagent (13778030, Invitrogen, Waltham, MA, USA), Lipofectamine 3000 (L3000001, Invitrogen), FuGENE transfection reagent (E2311, Promega, Sydney, Australia), Lipofectamine 2000 reagent (11668027, Invitrogen), Lipofectin reagent (18292011, Invitrogen) were used in this study. To evaluate the efficacy of transfection reagents to SSO, a 5′ end FAM labelled 24-mer scrambled ssDNA sequence (5′-FAM-CATCGATGGGAGCTCCGTGTCGTT-3′) was synthesized by Integrated DNA Technologies (IDT, Singapore). According to the BLAST analysis, this sequence does not match any sequence on human genome. The functional comparison assay used a miR21 targeting DNAzyme (5′-CATCGATGGGAGCTCCGTGTCGTT-3′). U87MG (human glioblastoma cells), Huh-7 and Hep G2 (human hepatocellular carcinoma cells), HEK293 (human embryonic kidney cells), and HL-60 (human leukemia cells) were purchased from Cell Bank Australia (Sydney, Australia). MCF-7 and MDA-MB-231 (human breast cancer cells) were purchased from ATCC, USA and kindly provided by Assoc Prof. Stacey Edwards at the QIMR Berghofer Institute, Brisbane, Australia; SH-SY5Y (human neuroblastoma cells) was kindly provided by Assoc. Prof. Bruno Meloni at the Perron Institute for Neurological and Translational Science, Perth, Australia; Human primary myoblast was kindly provided by Prof. Sue Fletcher and Prof. Steve Wilton at Molecular Therapy laboratory, Murdoch University, Perth, Australia, and JU77 (human lung mesothelioma cells) was purchased from Cell Bank Australia (Sydney, Australia) and kindly provided by Dr. Willem Lesterhuis, Harry Perkins Institute, University of Western Australia, Perth, Australia).

### 4.2. Cell Culture

U87MG and Hep G2 cells were cultured in Eagle’s minimum essential media (EMEM; ThermoFisher Scientific, Melbourne, Australia) supplemented with 10% FBS (F8192, Sigma, Sydney Australia). Huh-7, HEK293, MDA-MB231, JU77 and MCF-7 cells were cultured in Dulbecco’s Modified Eagle Media (12491-015, ThermoFisher) supplemented with 10% FBS. HL-60 was cultured in Roswell Park Memorial Institute medium (11875119, ThermoFisher) supplemented with 10% FBS. SHSY5Y was cultured in 45% EMEM supplemented with 10% FBS and 45% Ham’s F-10 (41550021, ThermoFisher). All cells were incubated at 37 °C in a humidified incubator supplying 5% CO_2_/air. 

### 4.3. SSO Transfection 

Cells were plated on a 24-well plate at 500 µL/well at density of 5 × 10^4^–10 × 10^4^ cells/mL (depending on cell conditions) in the indicated growth medium and propagated to 80% confluency at the time of transfection. The SSO was mixed with different transfection reagents and the complexes were prepared according to the manufacturer’s protocol. Solutions were combined, vortexed, and incubated for the appropriate time (according to specification of different reagents) to allow formation of complexes. In this study, 1 µg SSO was complexed with volume of 2 µL RNAiMAX, in the Opti-MEM Reduced Serum Medium (22600050, Thermofisher). 1 µg SSO was complexed with 2 µL of Lipofectamine 3000 [TR/DNA ratio (*w*/*w*) = 2:1] with 1.5 µL of P3000 as described in the manufacturer’s protocol, in the Opti-MEM Reduced Serum Medium. 1 µg SSO was complexed with 2 µL of FuGENE HD [TR/SSO ratio (*w*/*w*) = 2:1] in the Opti-MEM Reduced Serum Medium. 1 µg SSO was complexed with 2 µL of the Lipofectamine 2000 [TR/DNA ratio (*w*/*w*) = 2:1] in the Opti-MEM Reduced Serum Medium; 1 µg SSO was complexed with 2 µL of the Lipofectin [TR/SSO ratio (*w*/*w*) = 2:1] in the Opti-MEM Reduced Serum Medium. After 24 h incubation, the cells were washed with PBS, 500 µL transfection/SSO mixture was added to each well and incubated with the cells for 24 h at 37 °C in a humidified incubator supplying 5% CO_2_/air. All transfection assays were carried out in triplicate simultaneously for all five transfection reagents and with no reagent group as a control. 

### 4.4. Fluorescence Imaging

Twenty four hours after transfection using different transfection reagents and a FAM-tagged SSO (5′-FAM-CATCGATGGGAGCTCCGTGTCGTT-3′), 1 µL of Hoechst 33342 solution (1 µg/mL) was added to the plated cells and incubated for 10 min at 37 °C in a humidified incubator supplying 5% CO_2_/air. Media was aspirated and the wells were washed with 500 µL of 1× PBS a total of three times. To measure the effect of the background noise caused by non-specific cell surface binding of oligonucleotides, a fluorescence-quenching step was conducted according to our previous publication [10]. Simply, the surface fluorescence was quenched with 0.04% Trypan Blue (T8154, Sigma) for 3 min followed by thorough washing. Fluorescence microscopy was conducted using the Eclipse TS100 microscope (Nikon Australia; Sydney, Australia). To make the fluorescence density of different treatment groups comparable, the manual mode was used to image FAM labelled SSO, with a fixed 4 s exposure time applied. 

### 4.5. Relative Fluorescence Quantification

The quantification is conducted according to previously reported method [21]. Briefly, the integrated density (Intden), which displays both the area and mean signal values was used to depict the amount of transfected SSO. To compare the relative transfection efficacy of different transfection reagents in different cells, the Intden data was then normalized by cell numbers, which is denoted by the size area of Hochest33342 color (nucleic acid). Relative transfection efficacy = Intden _(SSO)_/Area _(cell nucleus)_. To facilitate comparison of efficacy of different reagents, the data were normalized by setting the highest fluorescence data as 100%.

### 4.6. Cell Viability Assay (MTT)

Cytotoxicity of the six transfection reagents were evaluated by MTT assay. Cells (3.5 × 10^4^ cells/mL) in 200 µL of indicated culture media were seeded in 96-well plates and incubated overnight. Treatment with transfection/SSO mix was conducted 24 h prior to the viability assay using MTT reagent using the same condition employed for transfection assay. Briefly, 5 mg/mL MTT reagent (M5655, Sigma) in 1× PBS (20 µL/well) was added into the plates and incubated for 3 h. After incubation, the medium was aspirated and dimethyl sulfoxide (150 µL/well) was added to stop the reaction. The optical density was quantified in a FLUROstar Omega multi-detection microplate reader (BMG Labtech, Offenburg, Germany) at 570 nm wavelength. The cell viability was calculated by comparing the luminescent signal of treatment group to the signal obtained with non-transfected control cells (setting as 100% viability). Each value represents the mean standard deviation from triplicates. 

### 4.7. Taqman qPCR to Measure the Expression of miR21 

Twenty four hours after transfection of a miR21 targeting DNAzyme (5′-CATCGATGGGAGCT CCGTGTCGTT-3′) using the same condition with the transfection assay, the total RNA of different treatments was harvested, and cDNA was prepared by TaqMan™ MicroRNA Reverse Transcription Kit (4427975, ThermoFisher) according to the supplier’s specification. The q-PCR was preformed using TaqMan Universal Master Mix (4440040, ThermoFisher) with RNU6B, RNU44, and RNU48 as internal controls. q-PCR was conducted using a C1000™ Thermal cycler, CFX96™ real-time system (BioRad, Sydney, Australia) and programmed initially at 95 °C for 10min, 95 °C for 15 s, then 60 °C for 1 min and repeated for a total of 40 cycles.

### 4.8. Statistical Analysis

All statistical analyses were performed using GraphPad Prism 3.03 (GraphPad Software, La Jolla, CA, USA). An unpaired *t* test was used for comparisons between two experimental groups. The relative transfection and cytotoxicity patterns of different transfection reagents were processed by the R program using the ggplot 2 Package (MathSoft, Cambridge, MA, USA). Unless otherwise indicated, all results were averaged from biological triplicates and values were reported as means ± SEM. A *p* value of less than 0.01 was considered statistically significant.

## Supplementary Materials

The supplementary materials are available online.
