# Supplementary material for: Systematic Screening of Commonly Used Commercial Transfection Reagents towards Efficient Transfection of Single-Stranded Oligonucleotides
Source: Molecules. 2018 Oct 8;23(10):2564. doi: 10.3390/molecules23102564 (PMC6222501; doi:10.3390/molecules23102564)
Supplement: Supplementary file 1 [file molecules-23-02564-s001.pdf]

molecules

*Article*

# Systematic Screening of Commonly Used Commercial Transfection Reagents towards Efficient Transfection of Single-Stranded Oligonucleotides

**Tao Wang[1,2†], Leon Larcher[1†], Lixia Ma[3], Rakesh N. Veedu[1,2*]**

[1] Centre for Comparative Genomics, Murdoch University, Perth, WA 6150, Australia
[2] Perron Institute for Neurological and Translational Science, Perth, WA 6009, Australia
[3] School of Statistics, Henan University of Economics and Law, Henan Province, 450046, China
[†] These authors contributed equally
* Correspondence: R.Veedu@murdoch.edu.au; Tel.: +61 8 9360 2803

Received: date; Accepted: date; Published: date

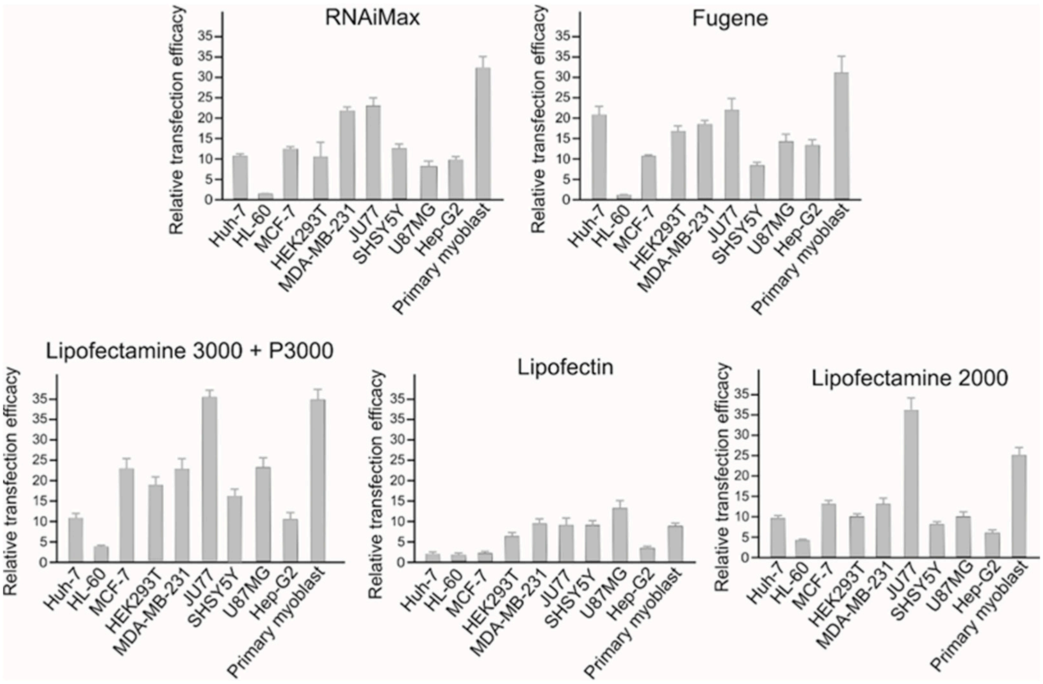


**Figure S1**. *In vitro* transfection efficiency of different transfection reagents in 10 established cells.
